# Incidence and main causes of severe maternal morbidity in São Luís, Maranhão, Brazil: a longitudinal study

**DOI:** 10.1590/S1516-31802011000300005

**Published:** 2011-05-05

**Authors:** Ana Paula Pierre Moraes, Sandhi Maria Barreto, Valéria Maria Azeredo Passos, Patrícia Silva Golino, Janne Ayre Costa, Marina Xerez Vasconcelos

**Affiliations:** I MD. Doctoral Student in the Public Health Program, School of Medicine, Universidade Federal de Minas Gerais (UFMG), Belo Horizonte, Minas Gerais, Brazil; II MD, PhD. Associate professor of the Postgraduate Public Health Program, School of Medicine, Universidade Federal de Minas Gerais (UFMG), Belo Horizonte, Minas Gerais, Brazil.; III MD, PhD. Associate professor of the Department of Clinical Medicine, School of Medicine, Universidade Federal de Minas Gerais (UFMG), Belo Horizonte, Minas Gerais, Brazil.; IV MSc. Obstetrician, Marly Sarney Maternity Hospital, São Luís, Maranhão, Brazil.; V MD. Resident in Gynecology and Obstetrics, University Hospital, Universidade Federal do Maranhão (UFMA), São Luís, Maranhão, Brazil.

**Keywords:** Pregnancy complications, Maternal mortality, Puerperium, Prenatal care, Pregnancy, Complicações na gravidez, Mortalidade materna, Puerpério, Cuidado pré-natal, Gravidez

## Abstract

**CONTEXT AND OBJECTIVE:**

Evaluation of severe maternal morbidity has been used in monitoring of maternal health. The objective of this study was to estimate its incidence and main causes in São Luís, Maranhão, Brazil.

**DESIGN AND SETTING:**

Prospective longitudinal study, carried out in two public high-risk maternity hospitals and two public intensive care units (ICUs) for referral of obstetric cases from the municipality.

**METHODS:**

Between March 1, 2009, and February 28, 2010, all cases of severe maternal morbidity according to the Mantel and Waterstone criteria were identified. The sociodemographic and healthcare characteristics of the extremely severe cases were compared with the less severe cases, using the Fisher, χ^2^, Student t and Mann-Whitney tests, with a significance level of < 0.05.

**RESULTS:**

127 cases of severe maternal morbidity were identified among 8,493 deliveries, i.e. an incidence of 15.0/1000 deliveries. Out of 122 cases interviewed, 121 cases were within the Waterstone criteria and 29 were within the Mantel criteria, corresponding to incidences of 14.1/1000 and 3.4/1000 deliveries, respectively. These rates were lower than those described in the literature, possibly due to case loss. The main causes were hypertension during pregnancy, which was more frequent in less severe cases (P = 0.001) and obstetric hemorrhage, which was more common among extremely severe cases (P = 0.01).

**CONCLUSIONS:**

Direct obstetric disorders were the main causes of severe maternal morbidity in São Luís, Maranhão. Investigation and monitoring of severe morbidity may contribute towards improving obstetric care in the municipality.

## RESUMO

**CONTEXTO E OBJETIVO:**

A avaliação da morbidade materna grave tem sido utilizada na vigilância à saúde materna. O objetivo deste estudo é estimar esta incidência e as suas principais causas em São Luís, Maranhão, Brasil.

**TIPO DE ESTUDO E LOCAL:**

Estudo longitudinal prospectivo, desenvolvido nas duas maternidades públicas de alto risco e nas duas unidades de terapia intensivas (UTIs) públicas de referência para casos obstétricos do município.

**MÉTODOS:**

Entre 1° de março de 2009 e 28 de fevereiro de 2010, foram identificados todos os casos de morbidade materna grave segundo os critérios de Mantel e Waterstone. As variáveis sociodemográficas e de atenção à saúde dos casos de extrema gravidade foram comparados aos casos de menor gravidade utilizando os testes de Fisher, χ^2^, t Student e Mann-Whitney, com nível de significância de 0,05.

**RESULTADOS:**

Foram identificados 127 casos de morbidade materna grave em 8.493 partos, gerando uma incidência de 15,0/1000 partos. Dos 122 casos entrevistados, 121 se incluíam nos critérios de Waterstone e 29 se incluíam nos critérios de Mantel, correspondendo a incidências de 14,1/1000 e 3,4/1000 partos, respectivamente. Estas taxas estão abaixo da descrita na literatura possivelmente por perda de casos. As principais causas foram a hipertensão na gravidez, mais presente nos casos de menor gravidade (P = 0,001) e a hemorragia obstétrica, mais comum nos casos de extrema gravidade (P = 0,01).

**CONCLUSÃO:**

As desordens obstétricas diretas são as principais causas da morbidade materna grave em São Luís, Maranhão. Investigar e monitorar a morbidade grave pode contribuir para a melhoria da assistência obstétrica no município.

## INTRODUCTION

Reduction of maternal morbidity is one of the United Nations Millennium Development Goals.^1^ The number of maternal deaths reflects the socioeconomic level and the quality of medical assistance, and is inversely related to the degree of human development. Maternal mortality is very unequally distributed among developed and developing countries, and the latter is responsible for 99% of all known maternal deaths in the world.^2^ Maternal mortality in Brazil is still high and was estimated as 77.2/100,000 live births in the year 2006, with differences between the more and less developed regions of the country.^3^

Increasing numbers of studies on maternal morbidity have been produced because maternal mortality rates do not reflect the quality of obstetric care accurately, due to the rarity of maternal death in developed countries.^4^ Population-based studies show that the burden of overall maternal morbidity is high and that monitoring of maternal morbidity is important for improving maternal health.^5^

Maternal morbidity refers to a continuum that begins with the occurrence of any complication during pregnancy, delivery or puerperium and ends with maternal recovery or death.

Studies on severe complications in obstetrics have introduced the term "near miss" into the medical sciences. This term was originally used by air traffic controllers to describe an air accident that was about to happen, but did not occur due to good judgment or luck.^6^ At first, severe maternal morbidity or near miss maternal morbidity related to women who presented potentially lethal complications during pregnancy, delivery or puerperium and who survived^7^ either due to access to hospital care or by chance.^8^ It was also defined as a life-threatening situation that would need urgent medical intervention to prevent possible maternal death.^9^ Women who lived through a near miss situation would probably present similar characteristics to those who died during pregnancy, delivery or puerperium, thus constituting a proxy model for maternal death.^10,11^

Studying severe maternal morbidity enables faster quantitative analysis and makes it possible to obtain information on the stricken woman herself, who can give detailed information about the treatment received and the care sought. Thus, such studies contribute towards monitoring the obstetric care network and process.^10^

There are distinct ways to define a case of severe maternal morbidity: 1) the complexity of care management, such as transfers to an intensive care unit (ICU)^12-14^ or carrying out hysterectomy;^15^ 2) the presence of maternal organic dysfunction, normally measured by means of the Mantel criteria, which provide comprehensive criteria for this definition;^8^ 3) the occurrence of certain diseases that are part of the pregnancy-puerperium cycle, referred to as the Waterstone criteria, which are the most cited representative of this definition;^16^ and 4) a sum of clinical and laboratory criteria based on organic dysfunction/organic failure, associated with the complexity of the care management, which has recently been defined as maternal near miss by the World Health Organization.^17^

Brazilian studies on the incidence of maternal morbidity have been carried out in Campinas, Goiânia and Recife, and these took into account the criterion based on ICU transfer^18-21^ and the Mantel and Waterstone criteria.^22-24^ The main causes for severe maternal morbidity in these studies were hypertension and obstetric hemorrhage.

Maranhão is one of the poorest states of Brazil. It has the lowest per capita income among the federal states and has one of the worst Human Development Indexes (HDI).^25^ The metropolitan area of São Luís (the state capital) has one of the worst HDIs in the country and has high maternal mortality.^26,27^ The city has around 18,000 deliveries per year, and more than 99% of them are in hospitals.^3^ Previous studies on severe maternal morbidity in São Luís were descriptive and were carried out in only one ICU.^28,29^

## OBJECTIVE

The present study aimed to estimate the incidence and main causes of severe maternal morbidity in São Luís, Maranhão, Brazil.

## METHODS

This prospective longitudinal study investigated the incidence of severe maternal morbidity based on the care provided under the Brazilian National Health System (Sistema Único de Saúde, SUS) in São Luís. The city's estimated population is one million inhabitants, and 85% of them only use the public healthcare system.

Information for this study was obtained from interviews carried out at two maternity referral ICUs out of the four available in the city and at the two high-risk maternity units, which are responsible for approximately 8,000 births per year, corresponding to half of the deliveries performed in the seven maternity hospitals of São Luís.

Data gathering took place between March 1, 2009, and February 28, 2010. Four trained medical researchers identified the cases through active searching of the medical records and direct verification with the health professionals at these institutions, at least three times a week.

The case definition of severe maternal morbidity followed the Mantel et al.^8^ and Waterstone et al.^16^ criteria. Every patient residing in the city and admitted to any of the study units due to complications during pregnancy, delivery or up to the 42^nd^ day of puerperium was classified as a case of severe maternal morbidity. Subsequently, the cases were classified into two subgroups: extremely severe maternal morbidity, which fulfilled the criteria of Waterstone and Mantel simultaneously; and other severe maternal morbidity, which fulfilled only the Waterstone criteria. This methodology followed preceding Brazilian studies.^22,24^

The incidence of severe maternal morbidity was estimated as the ratio of severe maternal morbidity cases per 1000 registered deliveries in the maternity hospitals investigated.

Information regarding sociodemographic factors, pregnancy and healthcare use were taken from the hospital and prenatal records when available. Further information on sociodemographic factors, pregnancy and the care received were also obtained from interviews with the patients, using a structured questionnaire at the hospital, after the subjects had signed a free and informed consent statement.

The data were entered using the Epidata software version 3.1. The statistical analysis was carried out using the Statistical Package for the Social Sciences (SPSS) software, version 15.0. The statistical significance of differences in proportions was tested using Pearson's chi-square test or Fisher's exact test. The normality hypothesis was tested by means of the Kolmogorov-Smirnov test for all continuous variables. Mean values were compared using Student's t test and medians using the Mann-Whitney test. The significance level was set at 0.05.

Investigations on maternal deaths are carried out systematically through the City Health Department of São Luís through an active search for deaths among women of fertile age. The numbers and descriptions of maternal deaths identified, according to causes and places over the same period as investigated in this study, were obtained from the City Committee of Infant and Maternal Mortality of São Luís by personal communication.

The present study was approved by the Research Ethics Committee of Universidade Federal de Minas Gerais (ETIC 589/08).

## RESULTS

During the study period, 8,493 deliveries occurred in the two high-risk maternity hospitals of São Luís and the ICUs included in this study. A total of 127 cases fulfilled the Mantel and Waterstone criteria for severe maternal morbidity in these services, corresponding to an incidence of 15.0 cases/1000 deliveries. Out of the 127 cases identified, three refused to participate in the study and two were lost because they left the hospital before the interview.

Among the 122 cases of severe maternal morbidity studied, 121 (99.2%) were within the Waterstone criteria, with an estimated incidence of 14.1 cases/1000 deliveries, and 29 (23.8%) were within the Mantel criteria, with an estimated incidence of 3.4 cases/1000 deliveries.

Out of all the cases studied, only one case of myocardiopathy was within the Mantel criteria of extreme severity but not within the Waterstone criteria. Among the 121 cases defined by the Waterstone criteria, 28 also fulfilled the Mantel criteria and were classified as extremely severe maternal morbidity (Table 1). Thus, 28 women were in the subgroup of extreme severity and 93 were in the subgroup named "other severe morbidity".

**Table 1.** Causes of severe maternal morbidity in São Luís, Maranhão, between March 1, 2009, and February 28, 2010 (n = 122)

Diagnosis on admission	n	%	Ratio/1000 deliveries
Hypertension	103	84.4	12.1
Severe preeclampsia	81	66.4	9.5
Eclampsia	14	11.5	9.5
HELPP syndrome	7	5.7	0.8
Preeclampsia superimposed on preexisting hypertension	1	1.6	0.1
Hemorrhage	14	11.4	1.6
Uterine atony	5	4.1	0.6
Placenta abruptio	5	4.1	0.6
Placenta previa	1	0.8	0.1
Uterine rupture	2	1.6	0.2
Ruptured ectopic pregnancy	1	0.8	0.1
Infected abortion	3	2.5	0.3
Myocardiopathy	1	0.8	0.1
Sepsis through appendicitis	1	0.8	0.1
Total	122	100.0	14.3

HELLP = hemolysis, elevated liver enzymes and low platelet count

No statistically significant difference was observed between the severity subgroups regarding the sociodemographic characteristics studied: age, ethnic group, income, educational level, length of time living at the current address and presence of a bathroom inside the home (Table 2).

**Table 2.** Distribution of women with maternal morbidity, according to severity subgroup and sociodemographic factors (n = 121). São Luís, Maranhão, 2009

	Other severe morbidity		Extremely severe morbidity		P-value
	n	%	n	%	
Age					0.27*
< 20	15	16.1	3	10.7	
20-34	57	61.3	22	78.6	
≥ 35 years	21	22.6	3	10.7	
Marital status					0.68†
Married	66	71.0	21	75.0	
Other	27	29.0	7	25.0	
Ethnic group					0.50*
White	9	9.7	5	17.9	
Dark	61	65.6	15	53.6	
Black	16	17.2	5	17.8	
Other	7	7.5	3	10.7	
Educational level					0.26†
< 8 years	20	21.5	7	25.0	
8-10 years	34	36.6	14	50.0	
≥ 11 years	39	41.9	7	25.0	
Income per capita					0.45*
< 0.25 MW (minimum wage)	28	30.1			
0.25 MW to < 0.5 MW	24	25.8	5	17.9	
0.5 MW to < 0.75 MW	19	20.4	11	39.2	
≥ 0.75 MW	18	19.4	5	17.9	
Not stated	4	4.3	7	25.0	
Length of time living time at current address					0.91†
< 1 year	19	20.4	6	21.4	
≥ 1 year	74	79.6	22	78.6	
Bathroom inside home					0.29*
Yes	82	88.2	27	96.4	
No	11	11.8	1	3.6	

* Fisher's exact test;

† Pearson χ^2^.

Histories of previous hypertension were significantly less frequent in the extreme severity group (Table 3). There were no significant differences in relation to the number of pregnancies, age at first pregnancy, number of prenatal consultations declared at the interview, previous abortions and referral to high-risk prenatal care.

**Table 3.** Distribution of maternal morbidity cases according to severity subgroup and pregnancy-related healthcare factors (n = 121). São Luís, Maranhão, 2009

	Other severe morbidity		Extremely severe morbidity		P-value
	n	%	n	%	
First pregnancy during adolescence					0.08*
Yes	39	41.9	17	60.7	
No	54	58.1	11	39.3	
Previous hypertension					0.02†
Yes	16	17.2	0	0.0	
No	77	82.8	28	100.0	
Number of pregnancies					0.86*
1	33	35.5	11	39.3	
2-3	39	41.9	12	42.9	
≥ 4	21	22.6	5	17.8	
Planned pregnancy					0.90*
Yes	21	22.6	6	21.4	
No	72	77.4	22	78.6	
Prenatal care					0.43†
Yes	87	93.5	25	89.3	
No	6	6.5	3	10.7	
Numbers of prenatal consultations*					0.09†
0-1	8	8.6	3	10.7	
2-3	10	10.8	7	25.0	
4-5	36	38.7	6	21.4	
6-7	25	26.9	9	32.1	
≥ 8	14	15.0	2	7.1	
No response				3.6	
Hypertension during pregnancy					0.09†
Yes	36	38.7	5	17.9	
No	51	54.8	20	71.4	
No prenatal assistance	6	6.5	3	10.7	
Previous abortion					0.44*
Yes	34	36.6	8	28.6	
No	59	63.4	20	71.4	
All prenatal procedures‡					0.45*
Yes	21	22.6	5	17.8	
No	71	76.3	22	78.6	
No response	1	1.1	1	3.6	
Referral to high-risk prenatal care					0.59†
Yes	34	36.6	8	28.6	
No	53	57.0	17	60.7	
No prenatal assistance	6	6.4	3	10.7	
Severe morbidity at first admission					0.13*
Yes	70	75.3	17	60.7	
No	23	24.7	11	39.3	
Admission to ICU					< 0.001†
Yes	0	0.0	16	57.1	
No	93	0.0	12	42.9	
Prenatal record available					0.05*
Yes	65	69.9	14	50	
No	28	30.1	14	50	

* Pearson χ^2^;

† Fisher's exact test;

‡ The procedures evaluated were: blood and urine laboratory tests, ultrasound, anti-tetanus vaccine and taking part in educational activities.

Prenatal record data were available for 79 women. There was no statistically significant difference between the women with and without prenatal records (Table 4). Based on these records, there were no statistically significant differences regarding the number of prenatal consultations, prenatal procedures, time between the last consultation and the delivery or the age at delivery, between the two severity subgroups analyzed. Hospital stay was significantly longer in the extremely severe group (Table 4).

**Table 4.** Distribution of 121 maternal morbidity cases according to severity and healthcare data during pregnancy, based on medical records (n = 121). São Luís, Maranhão, 2009

	Other severe morbidity		Extremely severe morbidity		P-value
	Mean (SD)	Median	Mean (SD)	Median	
Number of prenatal consultations	4.7 (2.0)	4.0	4.2 (1.8)	3.5	0.38*
Number of prenatal procedures	7.0 (8.6)	7.0	6.0 (2.7)	6.5	0.77†
Time between delivery and last consultation (days)	26.8 (35.9)	18.0	28.9 (39.7)	13.5	0.76†
Gestational age at delivery (weeks)	36.1 (3.3)	36.6	34.9 (4.7)	35.3	0.18*
Length of hospital stay (days)	8.4 (6.1)	6.0	16.0 (16.7)	13.0	< 0.001†

SD = standard deviation;

* Student's t test;

† Mann-Whitney test.

The primary causes of maternal morbidity in each subgroup are shown in Table 5. While hypertension was more present in the group of lower severity, hemorrhage was significantly more frequent in the extremely severe group.

**Table 5.** Primary causes of severity according to severity group of maternal morbidity

	Other severe morbidities		Extremely severe morbidities		P-value
	n	%	n	%	
Hypertension	85	91.3	18	64.3	0.001*
Hemorrhage	6	6.4	7	28.6	< 0.01*
Infections	2	2.2	3	10.7	0.23*

* Fisher's exact test.

During the study period, eleven maternal deaths were registered in the city of São Luís, and ten of these were directly linked to pregnancy. Among these ten deaths, five were related to hypertension, three related to abortion, one was caused by amniotic fluid embolism and one was caused by puerperal sepsis. The indirect cause of maternal deaths was: sepsis due to skin and subcutaneous infection (one case).^30^ None of these maternal deaths occurred in the high-risk maternity hospitals studied. Out of the four deaths that occurred in ICUs, which were all among cases referred from maternity clinics in the city, two were in the ICUs studied. Two deaths occurred at home and five in an emergency hospital without previous admission to a maternity hospital.

## DISCUSSION

The majority of the 122 cases of severe maternal morbidity in this study were young women (80% less than 35 years of age) and with very low monthly per capita income (56% less than half of the Brazilian minimum wage). Eighty percent of the women reported that they had not planned their pregnancy and over 90% had received prenatal assistance. Most of these women were admitted to hospital as cases of severe morbidity (71.9%), mainly due to hypertension during pregnancy. The average number of prenatal consultations (4.7) was slightly lower than the 5.5 consultations per delivery estimated by SUS Brazil in 2005,^31^ but lower than the goal of six prenatal consultations proposed by the Ministry of Health.

The incidence of severe maternal morbidity was estimated based on the most widely used criteria in the literature. The criteria for maternal near miss proposed by the World Health Organization^17^ were not evaluated in this work, because they were published after the beginning of the data gathering for the present study.

The Waterstone criteria comprise diseases that are part of the complications of the pregnancy-puerperium cycle and are the main causes of maternal death: hypertensive disorders, sepsis and severe hemorrhage. Even though these criteria are easily applied, they do not consider the woman's organic response, since they only include clinical criteria. Thus, they tend to include cases with a lower death risk than the criteria proposed by Mantel or WHO.^8,17^ The incidence of severe maternal morbidity in the present study, estimated as 14.1 cases/1000 deliveries, was within the range of the incidence rates reported in a recent systematic review (3.2 to 69.8/1000), based on clinical signs and symptoms.^32^ However, the incidence estimated in the present study was three times lower than that reported for Campinas (45/1000).^22,24^ This finding is surprising and suggests that there may have been some problem in ascertaining cases in the present work, because Campinas is a much wealthier city and has better healthcare indicators (i.e., it would be expected that São Luis would have a higher rate).

Part of the problem might be explained by poorer measurement and recording of blood pressure levels and proteinuria at the maternity hospitals included in this study. Lack of information on these two parameters would under-estimate the real number of cases in these services during the study period. But also, we cannot rule out the possibility that severe maternal morbidity cases based on the Waterstone criteria may have gone to other maternity hospitals in São Luís without further referral to the high-risk maternity hospitals or to the two ICUs included in this study.

Monitoring of obstetric disorders is very important for evaluating the quality of maternal assistance in developing countries.^33^ As shown in this work, the most frequent obstetric problems were the main causes of severe maternal morbidity and maternal death in São Luís. Consequently, the Waterstone criteria, and especially occurrences of eclampsia and HELLP syndrome, ought to be part of the longitudinal monitoring of severe maternal morbidity.

The results show that the incidence of severe maternal morbidity based on the Mantel criteria (3.4) was also below the figures described in the recent literature^32,34^ (between 3.8 and 101.7/1000 deliveries).

The Mantel criteria, which include 17 conditions of severe organic dysfunction or handling situations relating to these organic dysfunctions, are the ones most widely used to define situations of severe maternal morbidity. They take into consideration extreme conditions that normally result in admission to an ICU. A lower-than-expected incidence of severe maternal morbidity according to the Mantel criteria may be explained by a high maternal mortality ratio or case loss.

In order to clarify the hypothesis that there was a high maternal mortality rate over the study period, we obtained data by means of personal communication from the Maternal Mortality Committee of the Health Department of São Luís.^30^ The maternal mortality over the period was high and may explain, at least in part, why the maternal morbidity found in this study was lower than what one would expect based on data from other studies in the country.

However, attention is also drawn to the fact that none of the maternal deaths occurred in the two high-risk maternity hospitals studied. Among the four deaths in ICUs, only two were in the units studied. Two deaths occurred at home and five at the emergency sectors of general hospitals. While there were three deaths due to abortion, only one case of abortion was identified among the very severe morbidity cases identified. Thus, the low incidence of extremely severe maternal morbidity found in this study cannot be explained by high maternal mortality in the healthcare units studied. The results from the present analysis, in comparison with the information on deaths occurring in São Luís over the same period, suggest that cases of severe maternal morbidity are not being referred to the high-risk maternities as they should be. It appears that many patients with complications in the pregnancy-puerperium cycle are seeking assistance at the emergency sectors of general hospitals, since five out of the eleven maternal deaths registered over the study period occurred in such hospitals. It is thus advisable to verify this hypothesis by carrying out active surveillance of maternal morbidity cases at emergency sectors of general hospitals in São Luís. This will help to clarify whether and why the referral and counter-referral system is not working as it should.

These results are consistent with most studies in developing countries, which have shown that the majority of patients are admitted to hospitals in a critical condition,^35,36^ thus suggesting that there is a delay in reaching adequate medical assistance. This may be due to failure of the referral system for more complex levels or to delays by patients in seeking medical assistance. The high incidence of severe episodes at general emergency sectors suggests that there may have been failures within the healthcare system, with regard to identifying patients and starting appropriate treatment before the onset of a severely morbid event.^35^

Some studies on severe maternal morbidity in Brazil have also taken into consideration the criterion of admission to an ICU,^18,19^ with an incidence that ranged from 1.4 to 8.2/1000 deliveries. The incidence estimated in the present study (1.9/1000) is within this range. Although easier to apply, this criterion introduces considerable bias into the estimates, because it depends on service organization and availability of ICU beds.^3,2^

In São Luís, there is no obstetric ICU and the two high-risk maternity hospitals are physically separated from the general ICUs that support them. Moreover, Maranhão is one of the states with the worst ICU ratios (beds/population) in the country.^37^ This restricts indications to ICUs to the most extremely severe cases. In developed countries, one third of the cases of severe maternal morbidity are admitted to an ICU.^34,38^ In the present study, only 13% were admitted to the two referral ICUs of São Luís. Among the patients who presented organic dysfunction in accordance with the Mantel criteria, 43% were not admitted to an ICU. An emphasis on early detection of maternal risk and immediate referral to a more advanced care center with the possibility of admission to an ICU would ease the recovery from organic dysfunction and prevent multiple organ failure and maternal death.^39^

There were no statistical differences between the two subgroups of severe maternal morbidity regarding sociodemographic characteristics and the majority of the health-related variables, probably due to the great homogeneity of SUS users in São Luís. The higher frequency of previous hypertension in the lower severity subgroup and the significantly higher proportion of hemorrhage and longer hospital stay in the extremely severe morbidity subgroup were also found in other studies.^22,24^ These data indicate that hypertension control during pregnancy, as well as prevention and control of obstetric hemorrhagic syndromes, should be a priority in qualifying training among the obstetric staff in São Luís.

The present study presents important limitations. This study was restricted to the two high-risk maternity hospitals and to the two referral ICUs and did not represent all cases of severe morbidity in the city, since cases in other low or medium-risk maternity clinics may have occurred without being referred to the units investigated in this work. Furthermore, as the maternal death data suggest, some cases may have been seen at emergency sectors of general hospitals with no subsequent assignment to high-risk maternity hospitals.

With regard to the data, the information obtained from medical and prenatal records can vary in its completeness and quality. Moreover, regarding the information obtained through interview, it is possible that women who have experienced an event of extreme severity may answer differently from those who have faced a less severe event, even considering that they were both interviewed at the same time of the pregnancy-puerperium cycle and close to the time of hospital release.

## CONCLUSION

The present study shows that obstetric disorders are the main cause of severe maternal morbidity in São Luís, Maranhão. The incidence of severe maternal morbidity found in this study was lower than what was expected based on other Brazilian studies. This suggests that a significant number of cases are not being referred to high-risk maternity hospitals. This signals that there may have been important failures in the referral system relating to maternal care and that there is a need for deeper investigation of the path provided for pregnant women in need of specialized and emergency care in the municipality.
